# Validation of an algorithm to reveal the U wave in atrial fibrillation

**DOI:** 10.1038/s41598-018-30493-8

**Published:** 2018-08-09

**Authors:** M. S. Al-Karadi, A. J. Wilkinson, J. Caldwell, P. Langley

**Affiliations:** 1 Faculty of Science and Engineering, University of Hull, Hull, England UK; 2 Castle Hill Hospital, Hull & East Yorkshire NHS Trust, Hull, England UK

## Abstract

Major cardiac organisations recommend U wave abnormalities should be reported during ECG interpretation. However, U waves cannot be measured in patients with atrial fibrillation (AF) due to the obscuring fibrillatory wave. The aim was to validate a U wave measurement algorithm for AF patients. Multi-beat averaging was applied to ECGs of 25 patients during paroxysms of AF and the presence of U waves compared to those from the same patients during sinus rhythm (SR). In a further database of 10 long-term AF recordings, the number of beats for effective U wave extraction by the algorithm was calculated. U waves were revealed in all AF recordings and there was no significant difference between the presence of U waves in AF and SR (p = 0.88). U wave amplitude was significantly increased in AF (mean (s.d.) amplitude 55 (39) AF vs 37 (28) μV SR, p = 0.005). The presence of U waves could easily be discerned when as few as 10 beats were used in the algorithm. The study demonstrates the validity of the algorithm to reveal U waves in AF recordings. The algorithm offers the potential to detect U wave abnormalities in patients with AF.

## Introduction

The U wave is a small amplitude feature of the ECG occurring at the end of the T wave^1,2^. It is reported to be present in 90% of recordings in healthy subjects^1^ yet the genesis of the U wave is still in doubt^1,3,4^. The main hypotheses proposed include, i) delayed ventricular repolarisation of either Purkinje system or mid-myocardial cells and ii) stretch induced delayed after depolarisation caused by mechanoelectrical coupling^1,3–5^. However, none of the hypotheses is universally accepted^1–3,6^.

U wave abnormalities are linked with a range of cardiac diseases, such as ischemia and hypertension, or may be due to medications^2^. Major cardiac organisations such as American Heart Association (AHA) and Heart Rhythm Society (HRS) recommended that all U wave abnormalities should be reported during ECG interpretation^2,7^. Presently this is not possible for patients with the abnormal heart rhythm AF. Given that AF is associated with many cardiac disorders which are also associated with abnormal U waves, for example hypertension, and that AF patients are subject to a range of rate and rhythm control medications, it is essential to be able to monitor the U wave and detect U wave abnormalities in AF patients.

U waves have rarely been described in patients with AF. This is not surprising because the measurement is difficult due to the atrial fibrillatory wave obscuring the U wave^8^. The presence of U waves cannot be discerned in AF because the amplitude and morphology of the fibrillatory wave^9–11^ is similar to that of the U wave. Thus, it is important to develop methods which are capable of revealing the U wave in AF so that U wave abnormalities can be detected and reported for patients with this arrhythmia. Additionally, the ability to study U waves under the unique conditions presented by AF, i.e. the rapid beat interval changes and associated haemodynamic response, will facilitate the investigation of U wave mechanisms under such conditions which have never before been studied.

Although the U wave cannot be seen during AF, it is hypothesised that the U wave is present because U waves are readily seen in patients with paroxysmal AF during periods of SR. In a preliminary study we demonstrated the feasibility of revealing the U wave in AF using an algorithm based on ventricular beat averaging^8^. Signal averaging is a well-known signal processing technique used extensively to reduce noise when multiple repetitions of a small amplitude signal are available for measurement^12–14^. As such it allows small amplitude signal components to be measured in the presence of large amplitude noise but only when the signal and noise are uncorrelated^12–15^. In our application ‘signal’ refers to the U wave and ‘noise’ refers to the atrial fibrillatory activity and other unwanted components such as power line and electromyogram (EMG) artefact. By careful alignment and summation of increasing numbers of ventricular beats the amplitude of the uncorrelated noise is progressively reduced while the desired U wave is revealed in the resulting average beat. Theory predicts that the level of noise reduction achieved is a function of the number of averaged signal epochs^12–14,16^, so the quality of the resulting U wave depends on the number of beats used to generate the average beat.

The aims of the present work were two-fold:

(i) First, to validate the beat averaging algorithm. Our preliminary study^8^ provided no validation of the U wave extraction algorithm, so the first aim was to validate the algorithm. Here we validate the algorithm by comparing U waves extracted from AF recordings to U waves readily observable in SR recordings for the same patients. This is referred to as the *‘validation study’*. Assuming U waves are unchanged during AF, validation implies the presence of U waves in both AF and SR recordings.
(ii) Second, to define the requirements for effective U wave extraction in terms of the number of beats needed for beat averaging. Since the quality of the extracted U wave is dependent upon the number of beats used to generate the average beat, the second aim was to establish the number of beats required for effective U wave extraction in AF recordings. This is referred to as the *‘number of beats study’*. It has important implications for the clinical application of the algorithm since the number of beats determines the ECG recording duration.

## Results

### Validation study

From the database of 25 patients for which recordings in both AF and SR were available, 3 patients were excluded because fast heart rates during SR caused the U waves to be obscured by following P waves in these patients. After applying the beat averaging algorithm to the recordings of the remaining 22 patients all 22 patients had visible U waves in both AF and SR. There were no discrepancies between the reporting of the presence of U waves by the two observers. Table 1 provides the contingency table for U waves extracted from AF and those in SR. There was no significant difference in the presence of U waves in AF compared to SR (p = 0.88).

**Table 1:** Contingency table of presence of U waves in AF and SR for 22 subjects of the validation study.

ECG	Visible U wave		Total
	Yes	No	
AF	22	0	22
SR	22	0	22
Total	44	0	44

**Figure 1:**
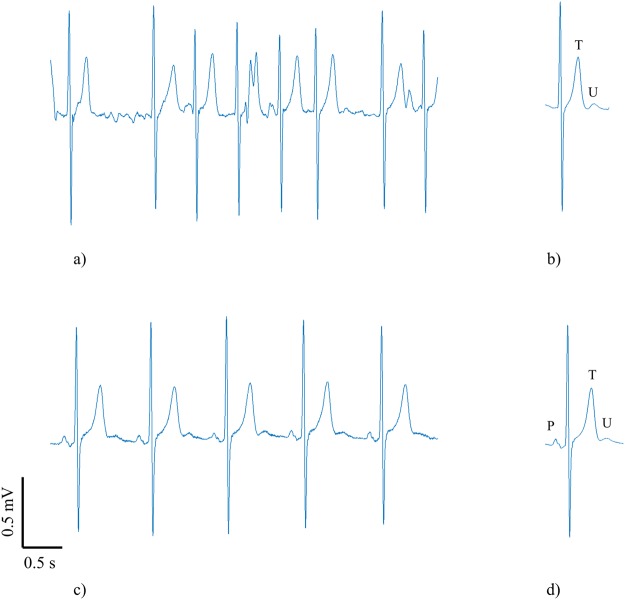
Example to illustrate the presence of U waves in a patient in both AF (top row) and SR (bottom row). (**a**) 5 s strip of ECG in AF in which it is impossible to discern the U waves and (**b**) the corresponding extracted average beat in AF with clear U wave following the T wave. (**c**) 5 s strip of ECG of the same patient during SR demonstrating clear U waves at each beat and d) the corresponding average beat in SR showing a clear U wave with the same morphology as those in (**b**,**c**).

Figure 1 shows a representative example of U waves extracted from AF and SR recordings for the same subject. Figure 1a illustrates the difficulty of discerning the U wave in AF. It shows a 5 second strip of ECG in which the irregular beat intervals and atrial fibrillatory wave obscure the underlying U wave. The U wave was revealed by the beat averaging algorithm as shown in Fig. 1b where the U wave extracted from the AF recording is clear and, as expected, follows the T wave. Further validation of the algorithm is provided in Fig. 1c,d which show the 5 s strip and average beat respectively for the same patient but in SR. The U wave extracted by the algorithm in AF was of the same morphology to that in SR but with increased amplitude.

**Figure 2:**
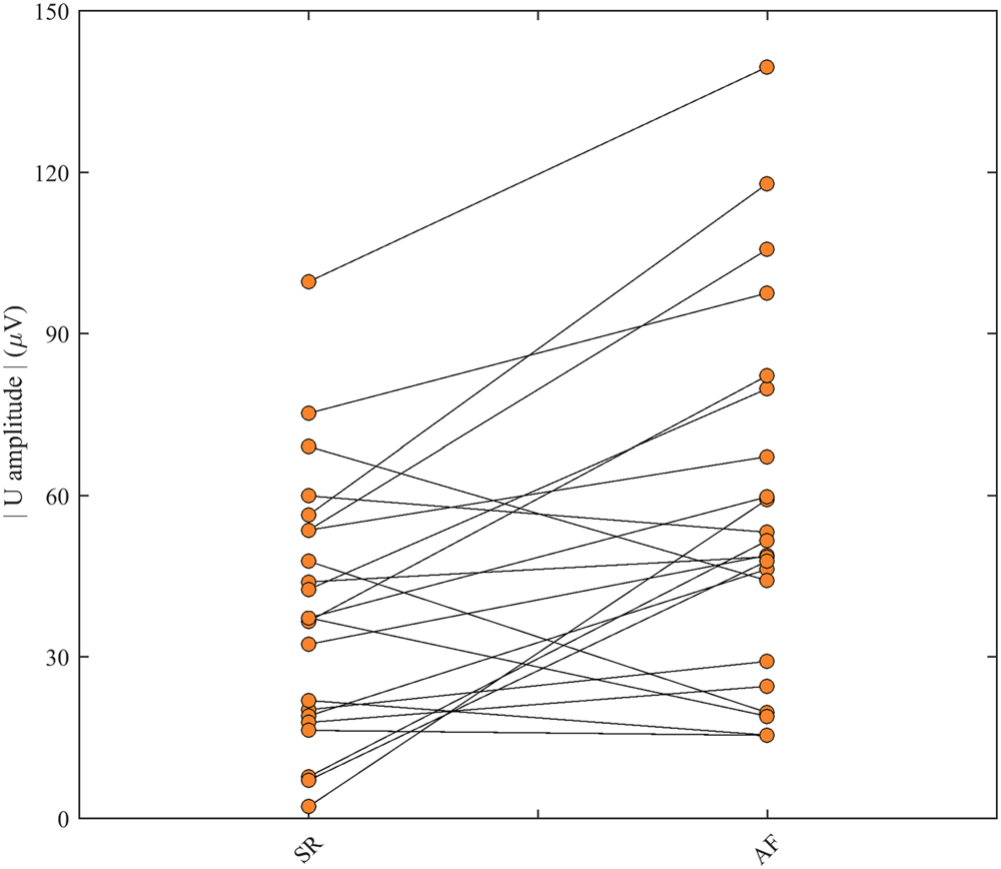
Comparison of U wave amplitudes measured during SR and AF for the 22 patients in the validation study.

Patients exhibited a wide range of U wave amplitudes. Figure 2 shows the paired relationship between U wave amplitudes in SR and AF recordings. The patient with the largest U waves in SR recordings also had the largest U waves in AF recordings but the mapping was not consistent across all patients. Interestingly, U wave amplitudes during AF were significantly larger than during SR (mean (s.d.) 55 (39) vs 37 (28) μV, p = 0.005) with the majority of patients (73% (16/22)) exhibiting larger U waves during AF compared to SR.

### Number of beats study

**Figure 3:**
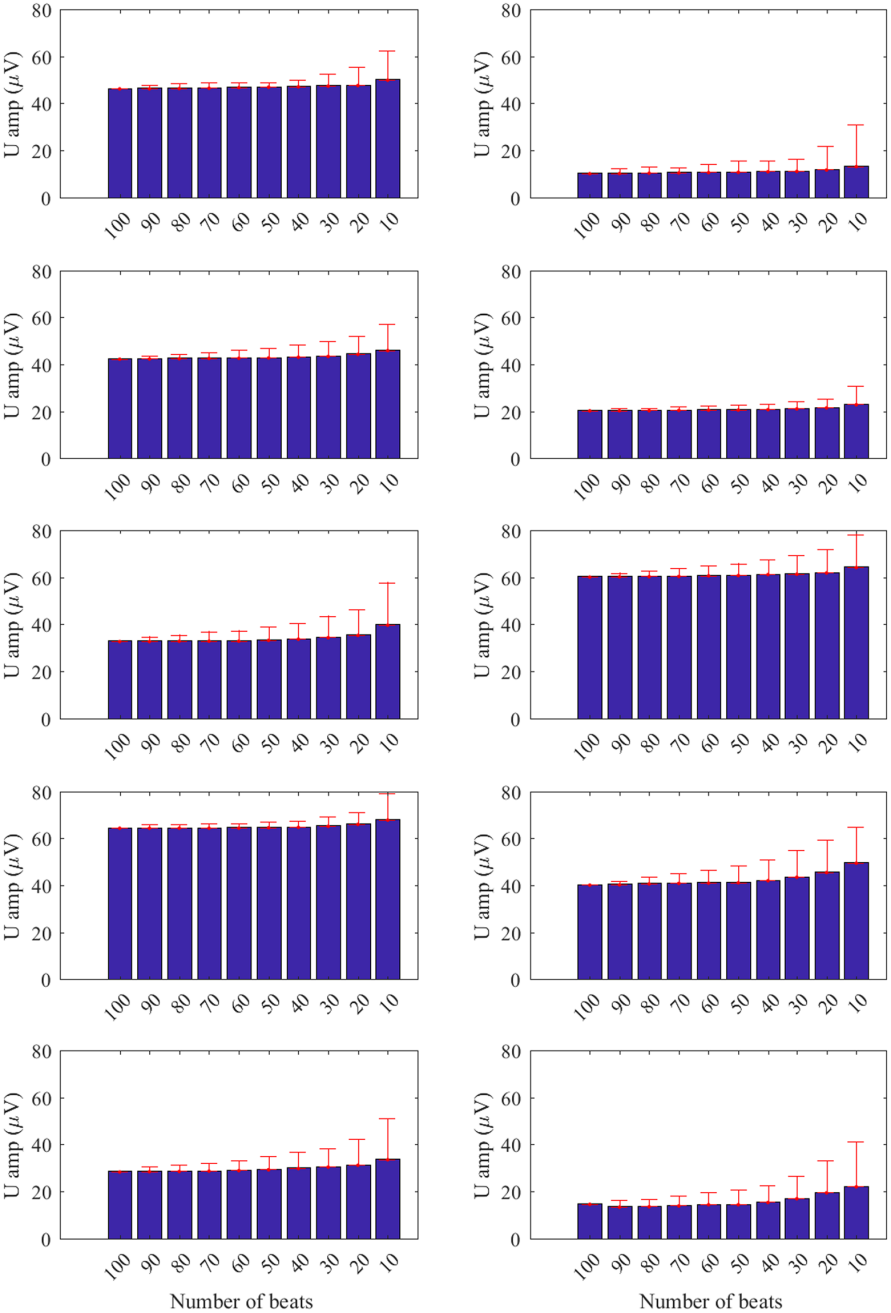
U wave amplitudes (U amp) for 10 patients in AF, one subplot for each patient. The effect on U wave amplitude by decreasing the number of beats (from 100 to 10) used to generate the average beat is illustrated. Bars and whiskers indicate mean and standard deviation of amplitude across the 10 sub-trials for N_beats_ = {90, 80, 70, 60, 50, 40, 30, 20, 10}.

Figure 3 shows the effect on U wave amplitudes by changing the number of beats (N_beats_) used to generate the average beat in the U wave extraction algorithm for all recordings. The effect of reducing the number of beats from the maximum of 100 beats (reference, N_beats_ = 100) was to increase the U wave amplitude in all cases. The amplitude increase can be explained by the presence of increasing levels of noise contaminating the extracted U wave as the number of beats is reduced. Theory predicts that as the number of beats is reduced the effectiveness of noise reduction by beat averaging decreases.

**Figure 4:**
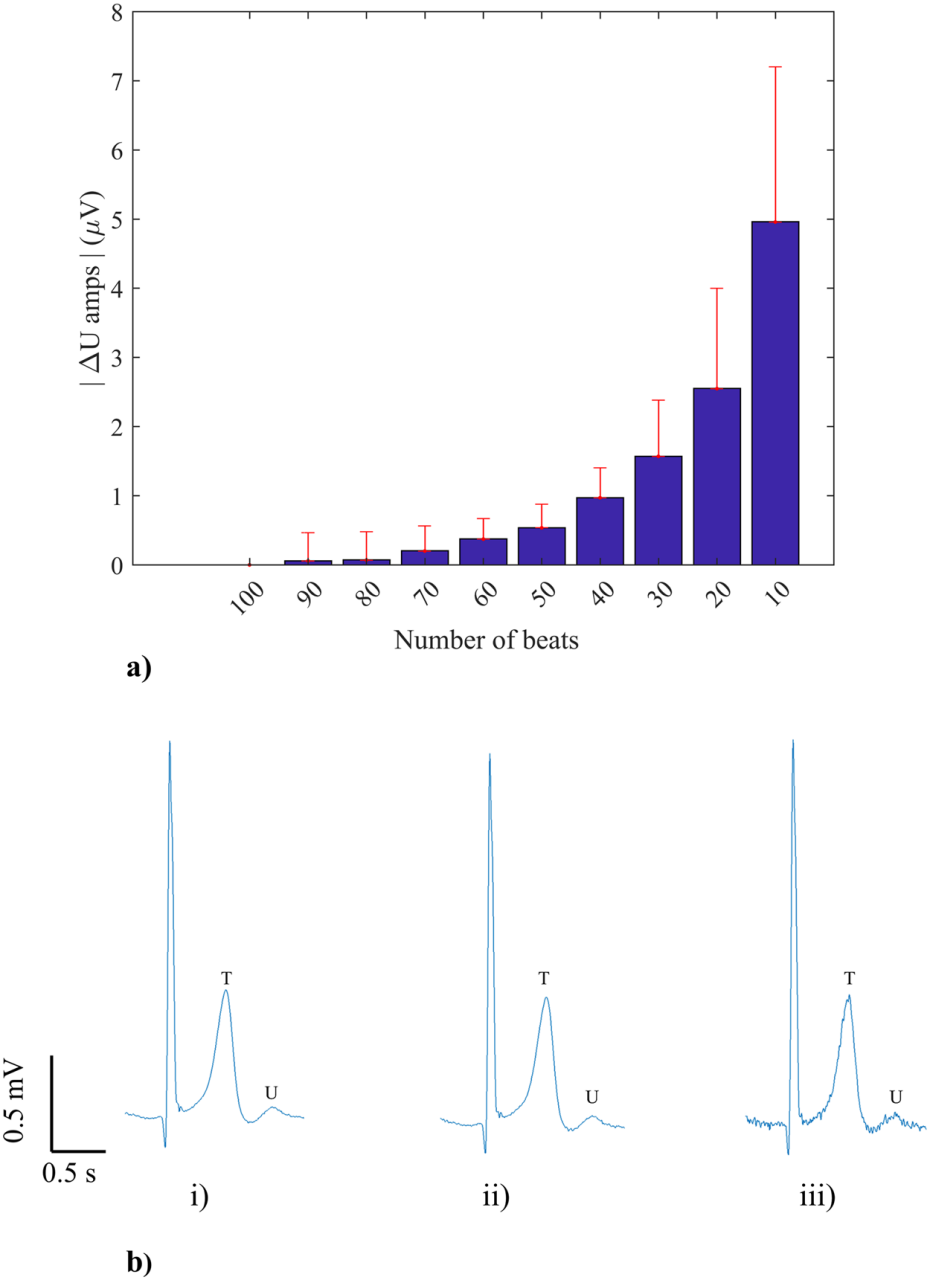
(**a**) The difference in U wave amplitude (ΔU amp) relative to reference amplitude (N_beats_ = 100) for reducing numbers of beats (N_beats_ = 90…10). Bars and whiskers indicate mean and standard deviation of amplitude difference across the 10 patients. (**b**) Average beats generated from the same AF recording using (i) 100 beats, (ii) 70 beats and (iii) 10 beats. Increased noise level can be seen in (iii) but the presence of a U wave is readily apparent.

Combining the data for all subjects and analysing the difference of U wave amplitude (ΔU amp) relative to the reference U wave amplitude, Fig. 4a shows that there was a mean amplitude difference of 5 μV when using 10 beats rather than 100 beats to generate the average U wave. In other words, residual noise increased to 5 μV when only 10 beats are used in the algorithm compared to 100 beats. Mean amplitude differences were significantly greater than zero across the trials (p = 0.0000183, within-subject ANOVA) and post-hoc analysis suggested that there were no significant differences in amplitude with respect to reference when at least 70 beats were used (mean (s.d.) ΔU amp = 0.2 (0.4) μV, N_beats_ = 70 beats, p = 0.1063). When expressed as a percentage of the U wave reference amplitude the mean amplitude increase was less than 1% when using 70 or more beats and increased to 14% when using only 10 beats. Nonetheless, it was observed that with as few as 10 beats (N_beats_ = 10) the presence of U waves could readily be visually discerned as illustrated in Fig. 4b.

## Discussion

This is the first study which validates an algorithm to reveal the U wave in AF patients. In the ‘validation study’ U waves were present in all 22 patients during episodes of AF for which U waves could be seen in corresponding SR recordings in the same patients. U waves in AF had the same polarity and morphology as those in SR but with increased amplitude in most subjects. The increased amplitude in AF could not be accounted for by residual noise (i.e. residual atrial fibrillatory wave) since none could be seen in the resulting average beats after close visual inspection and as illustrated in the example shown in Fig. 1. With the ability to now measure U waves during AF the algorithm provides the opportunity for researchers to explore potential mechanisms which might explain differences in U waves during AF and SR.

**Figure 5:**
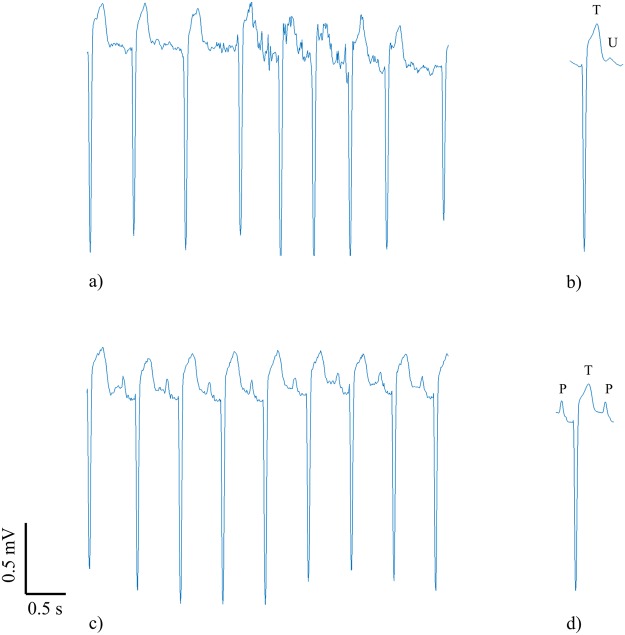
Example to illustrate the presence of U waves in a patient in AF (top row) and the apparent absence of U wave in SR (bottom row). (**a**) 5 s strip of ECG in AF in which it is impossible to discern the U waves and (**b**) the corresponding extracted average beat in AF with clear U wave following the T wave. (**c**) 5 s strip of ECG of the same patient during SR with no visible U waves at each beat and (**d**) the corresponding average beat in SR also showing no U wave. The U wave in SR is likely hidden by the following P wave.

Interestingly, when we applied our algorithm to the AF recordings of the 3 patients which had no observable U wave in SR, U waves were present during AF as illustrated in the example of Fig. 5. Although U waves could not be seen in the 3 SR recordings it seems likely that U waves were present but simply obscured by the P waves of the next cardiac cycle due to the fast heart rates in these SR recordings. Surawicz stated that the U wave cannot be seen at fast heart rates^1^.

Although the mechanism of U wave genesis is unknown, the U wave is a reliable feature unvarying in its occurrence at the end of the ventricular cycle in every ventricular beat. The algorithm exploits this feature by using the beat averaging technique to reveal the low amplitude U wave^12–14^. By averaging a number of ventricular beats the overall noise components in the ECG signal are reduced^12–14^. It is effective because the U wave and noise, which includes the atrial fibrillatory wave, are uncorrelated. The noise is effectively ‘averaged out’ over a number of ventricular beat cycles.

The number of beats to use in the calculation of the average beats is an important consideration since the greater the number of beats the greater the noise reduction. On the other hand, the greater the number of beats, the longer duration of recording which is required with associated resource implications. The ‘number of beats study’ showed that decreasing the number of beats from 100 to 70 did not significantly affect the U wave amplitude. However, the U wave was readily observable when the average beat was generated from as few as 10 beats with an average increase in noise amplitude of only 5 μV or 14% of reference amplitude. The study can be used to guide the requirement for the number of beats to use dependent upon the required application.

While in SR recordings the beat averaging algorithm removed some of the noise inherent in all clinical ECG recordings, the U wave can be seen without beat averaging in SR with good recording technique. However, at high heart rates the U wave becomes obscured by the following P wave^1^.

The algorithm satisfies an unmet clinical need because until now it has not been possible to report U waves in AF. Many studies have noted the diagnostic value of U wave abnormalities, particularly inverted U waves^17–19^. Although we did not detect any abnormal U waves in our cohort the algorithm facilitates the reporting of U wave abnormalities in AF patients as recommended by the important cardiac associations^2,7^. Further, the algorithm can be used to provide mechanistic insight into the origin of the U wave since AF is unique in its rapidly changing ventricular beat intervals. Preceding RR interval is known to affect ventricular filling dynamics^20,21^ which are implicated in the genesis of the U wave through the electro-mechanical hypothesis of the U wave^1,4,5,7,22^.

In conclusion, ventricular beat averaging reveals the U wave in AF patients. The algorithm facilitates the detection and reporting of U waves and their abnormalities in AF patients.

## Methods

### ECG databases

Two ECG databases were used in this work according the requirements of the sub-studies as described below. The study was approved by the University of Hull and Northumberland Local Research Ethics committees and all methods were performed in accordance with the relevant guidelines and regulations.

### Validation study

The database comprised recordings from 25 patients for which both AF and SR recordings in the same patient were available. Fourteen suitable recordings were available from the PAF Prediction Challenge database from PhysioNet^23^. A further 11 historical recordings from routinely collected ECG during electrophysiological studies at Castle Hill Hospital were used. The requirement for individual patient consent was waived as the study did not impact clinical care and all data were anonymised. Recording duration for AF or SR was a minimum of 4 minutes and recordings had sampling rate of either 128 Hz (PhysioNet) or 1953Hz. The PhysioNet recordings comprised two unspecified leads^23^ whereas the others had standard 12-lead ECG. As the lead with the most prominent U wave^8^ lead V4, or if V4 was not available (i.e. PhysioNet recordings) the lead with the most prominent U wave, was analysed.

### Number of beats study

The requirement for this database was the availability of long duration AF recordings to ensure a large number of ventricular beats to be used in the beat averaging algorithm. For this purpose, an existing database of 15 minute, 12-lead ECG recordings from 10 AF patients was used. Patients gave informed consent for these recordings. It is the same database used in our preliminary study^8^. The sampling rate was 500 Hz and lead V4 was analysed in this study.

### U wave extraction algorithm

#### AF recordings

**Figure 6:**
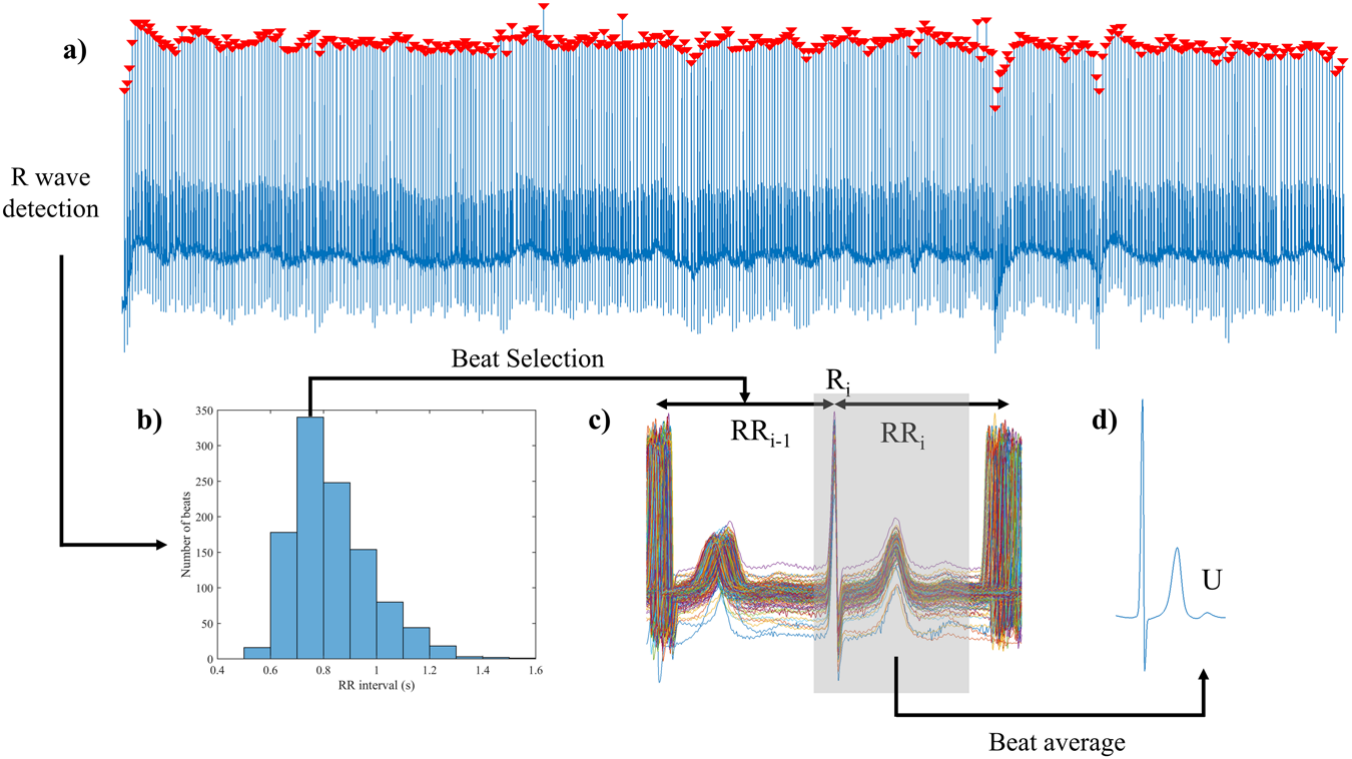
Signal processing workflow to extract U waves from AF recordings. R wave peaks () were detected in the ECG lead (**a**) from which the RR histogram (bin size RR_bin_ = 100 ms) was created (**b**). Beats meeting the selection criteria (RR_i−1_ = RR_mode_ ± 50 ms and RR_i_ > RR_min_) were extracted from the ECG lead and aligned to the R peak (R_i_) (**c**). The average beat (**d**) was calculated from the collection of beats over the interval corresponding to the grey area in (**c**) revealing the U wave.

U waves were extracted using a beat averaging algorithm. The average beat was formed using only beats with similar preceding RR interval. Careful selection of qualifying beats with respect to preceding RR interval and their alignment ensured optimum U wave extraction and minimised the effects of heart rate dependency of U wave timing and amplitude. The ECG processing workflow is illustrated in Fig. 6 and comprises (i) R wave detection, (ii) RR histogram, (iii) selection of qualifying beats and (iv) calculation of the average beat and identification of the U wave.

R wave peaks (R_i_) were detected automatically and confirmed by visual inspection (Fig. 6a). If present, ectopic beats and their adjacent intervals were removed. RR intervals were calculated (RR_i−1_ = R_i_ − R_i−1_). The RR histogram with bin size (RR_bin_) of 100 ms was constructed so that the number of beats (N_beats_) at specific RR intervals could be identified (Fig. 6b). To ensure the average beat was generated using the maximum number of beats with similar RR intervals, only beats falling within the bin with the most number of beats were selected (Fig. 6c). In other words, qualifying beats had RR_i−1_ = RR_mode_ ± 50 ms, where RR_mode_ was the histogram bin with most number of beats. A further requirement was to exclude beats with short following RR intervals (RR_i_) that would otherwise contaminate the averaged U wave, therefore beats with RR_i_ less than a threshold (RR_min_) of 650 ms were excluded. All qualifying beats were aligned to their R wave peak (R_i_) (Fig. 6c), and the average beat in each lead was calculated as the mean amplitude across the beats (Fig. 6d). The presence of a U wave was identified from the average beat (Fig. 6d). It is important to note that it is the preceding RR interval (RR_i-1_) which is important to consider rather than the RR interval containing the U wave (RR_i_) since our preparatory work indicated that the preceding RR interval is the major determinant of U wave amplitude.

#### SR recordings

With good quality SR recordings, the U wave can be readily seen without significant processing. However most clinical recordings present some noise and by applying the same beat averaging algorithm as for the AF recordings the resulting noise reduction allows for optimal presentation of U waves. Hence SR recordings were processed by the same algorithm used for the AF recordings. However, in SR at fast heart rates the P wave can impinge on the U wave so it was necessary to extend the RR_min_ interval to ensure that any following P waves would not contaminate the beat averaged U wave.

### Data analysis

#### Validation study

For each patient the presence or absence of U waves in the average beat was assessed visually and independently by two observers (MSA, PL). A positive validation outcome was defined as the presence of U waves of same polarity and morphology in both SR and AF recordings from the same patient, otherwise a negative validation outcome was recorded. Validation outcomes were recorded in a contingency table and statistical significance of agreement between U wave presence in AF and SR assessed by McNemar’s test. U wave amplitude from baseline to U peak was measured automatically but confirmed visually. Stable baseline amplitude was estimated from a 10-sample window in the electrically quiescent period before the onset of the QRS complex in AF recordings and before the onset of the P wave in SR recordings. Significance of differences in amplitude between SR and AF recordings was assessed by paired t-test.

#### Number of beats study

To determine the effect of the number of beats (N_beats_) on the quality of extracted U waves in AF recordings, the algorithm was systematically iterated with diminishing number of beats used for beat averaging at each iteration. Trials were conducted where the number of beats was systematically reduced from 100 beats (reference) in steps of 10 down to 10 beats (i.e. N_beats_ = {100, 90, 80, 70, 60, 50, 40, 30, 20, 10}).

Rather than discard some beats for trials where N_beats_ was less than 100, multiple sub-trials were conducted so that all 100 beats were analyzed across all the sub-trials. This was achieved by dividing the 100 beats into contiguous groups of 10 beats. For each N_beats_ trial, 10 sub-trials were conducted, systematically including the appropriate number of groups of 10 beats so that over the 10 trials all the beats were used. See the example in Figure A1 (supplementary document).

Noise reduction and hence U wave quality was quantified by U wave amplitude since U waves contaminated by noise have larger amplitude than clean U waves. Amplitude measurement was as described for the validation study. Differences between U wave amplitude for each N_beats_ trial and reference U wave (N_beats_ = 100 beats) were calculated as ΔU amp = U amp (N_beats_) − U amp (N_beats_ = 100)) and statistical differences with respect to zero amplitude difference assessed by within subjects ANOVA and post-hoc t-test. All tests were two-sided and p-values less than 0.05 considered statistically significant.

## Electronic supplementary material

Supplementary Material

## Data Availability

The datasets generated and analysed during the study are available from the corresponding author upon reasonable request.
