# Supplementary material for: Validation of an algorithm to reveal the U wave in atrial fibrillation
Source: Sci Rep. 2018 Aug 9;8:11946. doi: 10.1038/s41598-018-30493-8 (PMC6085295; doi:10.1038/s41598-018-30493-8)
Supplement: Supplementary file 1 — Supplementary Material [file 41598_2018_30493_MOESM1_ESM.docx]

**Validation of an algorithm to reveal the U wave in atrial fibrillation**

MS Al-Karadi^1^, AJ Wilkinson^1^, J Caldwell^2^, P Langley^1*^

^1^ Faculty of Science and Engineering, University of Hull, Hull, England, UK

^2^ Castle Hill Hospital, Hull & East Yorkshire NHS Trust, England, UK

* p.langley@hull.ac.uk

**Supplementary Document**


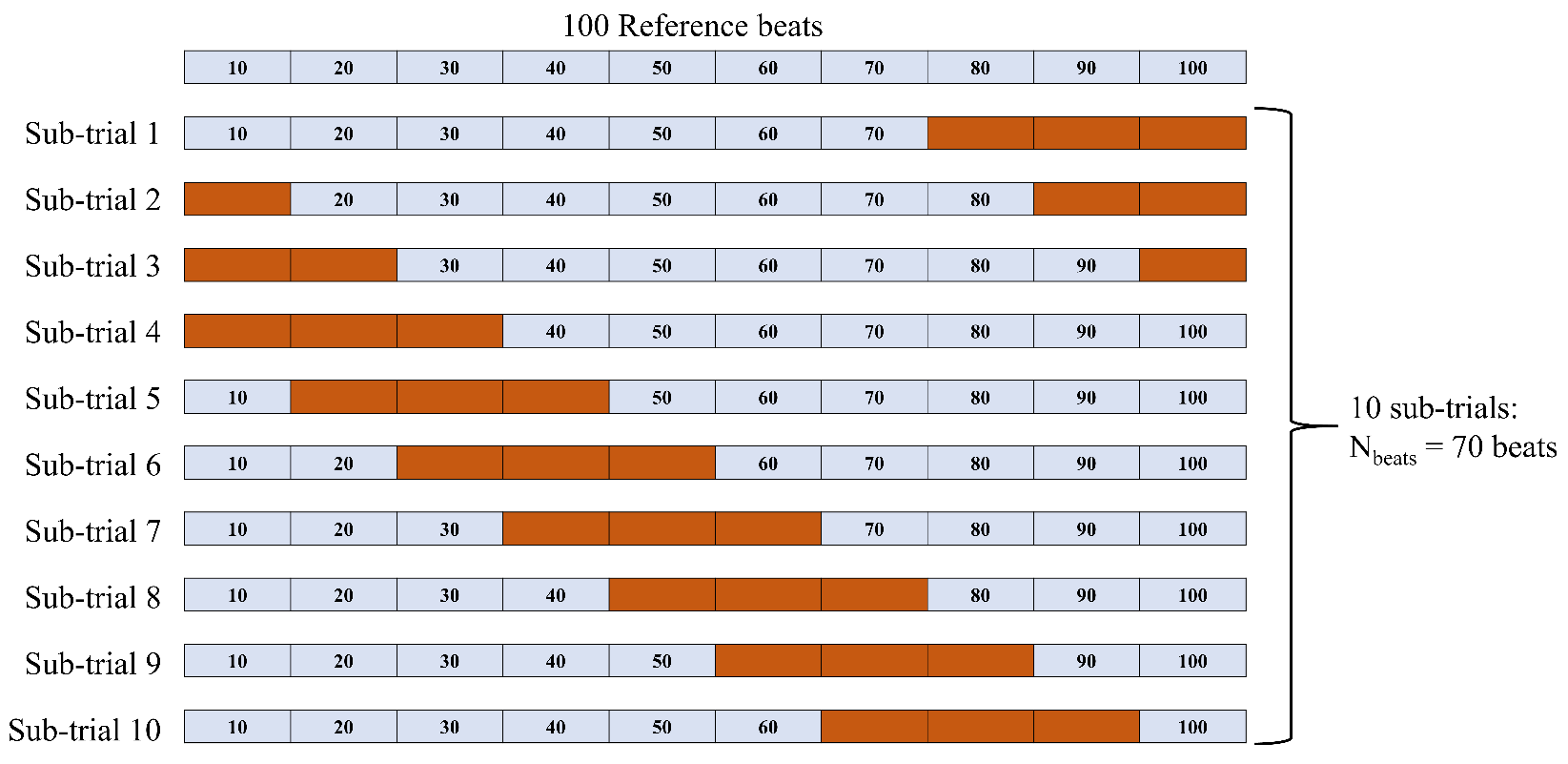


**Figure A1**. Illustration of the selection of beats from the 100 reference beats used in the 10 sub-trials when the number of beats in the trial was 70 beats (N_beats_ = 70 beats). Each sub-trial generated an average beat so for each trial of N_beats_ = {90, 80, 70, 60, 50, 40, 30, 20, 10}, there were 10 sub-trials generating 10 U waves which were compared to the reference U wave. The U waves from the average beat using all 100 beats (N_beats_ = 100) served as the ‘gold-standard’ reference U wave since it benefited from the greatest level of noise reduction.
